# Analysis of regulating activities of 5′-epiequisetin on proliferation, apoptosis, and migration of prostate cancer cells *in vitro* and *in vivo*

**DOI:** 10.3389/fphar.2022.920554

**Published:** 2022-08-10

**Authors:** Xueni Wang, Xiaowei Luo, Xia Gan, Chunmei Chen, Zaizhun Yang, Jing Wen, Wenxuan Fang, Hailing Huang, Chenghai Gao, Xuefeng Zhou, Xiaotao Feng, Yonghong Liu

**Affiliations:** ^1^ CAS Key Laboratory of Tropical Marine Bio-resources and Ecology/Guangdong Key Laboratory of Marine Materia Medica, South China Sea Institute of Oceanology, Chinese Academy of Sciences, Guangzhou, China; ^2^ Institute of Marine Drugs, Guangxi University of Chinese Medicine, Nanning, China; ^3^ Guangxi Zhuang Yao Medicine Center of Engineering and Technology, Guangxi University of Chinese Medicine, Nanning, China; ^4^ Guangxi Key Laboratory of Chinese Medicine Foundation Research, Nanning, China

**Keywords:** 5′-epiequisetin, prostate cancer, phosphoinositol 3-kinase, protein kinase B, death receptor 5

## Abstract

Advanced prostate cancer has a poor prognosis, and it is urgent to develop new effective drugs. 5′-Epiequisetin is a tetramic acid derivative which was isolated from a marine sponge-derived fungus *Fusarium equiseti* in our previous study. In this study, 5′-epiequisetin showed cytotoxicity against four prostate cancer cell lines, namely, LNCaP, 22Rv1, DU145, and PC-3 cells, with the lowest IC_50_ value of 4.43 ± 0.24 μM in PC-3 cells. Further studies showed that it could dramatically regulate the clonal colony formation, apoptosis, and migration of PC-3 cells. In addition, flow cytometry data showed that 5′-epiequisetin could block the cell cycle at the G1 phase. Proteome profiler array and Western blot revealed that 5′-epiequisetin could regulate the expression of proteins responsible for cell proliferation, apoptosis, and migration. 5′-Epiequisetin regulated the expression of PI3K, Akt, phosphorylated Akt, and proteins which control the cell cycle. Meanwhile, 5′-epiequisetin upregulated expression of DR5 and cleave-caspase 3, which play important roles in the process of apoptosis. Moreover, when DR5 was silenced by small interfering RNA, the proportion of apoptotic cells induced by 5′-epiequisetin remarkably declined. In addition, 5′-epiequisetin downregulated the expression of survivin which plays a key role in the process of survival and apoptosis. 5′-Epiequisetin also impacted beta-catenin and cadherins, which were associated with cell migration. In addition, 5′-Epiequisetin significantly inhibited the progression of prostate cancer in mice, accompanied by regulating the protein expression of DR5, caspase 8, survivin, and cadherins *in vivo*. Taken together, these findings indicated that 5′-epiequisetin showed an anti–prostate cancer effect by inducing apoptosis and inhibiting cell proliferation and migration both *in vitro* and *in vivo*, suggesting a promising lead compound for the pharmacotherapy of prostate cancer.

## Introduction

Global cancer statistics 2020 showed that prostate cancer (PCa) ranked the second highest incidence rate in men, and the American cancer society predicted that PCa would be the most common cancer among men in the United States in 2021 (Siegel et al., 2021; Sung et al., 2021). In China, PCa is the sixth most common cancer in males (Zhang S. et al., 2021). Patients with localized prostate cancer could get an optimistic prognosis after taking a standard therapeutic regimen, while patients with locally advanced or extensive metastasis will eventually develop castration-resistant prostate cancer (CRPC) (Ge et al., 2020). In the past decades, remarkable progress has been made in the treatment of CRPC, and the 5-years survival rate of patients with CRPC has been improved by therapies such as immunotherapy, hormonal therapy, cytotoxic therapy, targeted therapy, and bone-directed therapy (Galsky et al., 2012). However, there is still an urgent need to find more advanced therapies to overcome insurmountable problems, such as drug resistance and adverse reactions.

Marine natural products (MNP) have been evidenced as an important source of lead compounds with novel chemical structures (Newman and Cragg, 2020; Carroll et al., 2021). An increasing number of marine-derived anti-tumor drugs have been introduced into the market; meanwhile, there are also many marine-derived agents in various stages of clinical trials (Mayer, 2020). It is worth noting that dozens of MNPs have been found with an anti-PCa effect based on unique mechanisms of action (Fan et al., 2018; Dyshlovoy and Honecker, 2020). For instance, elaiophylin and Ilicicolin A are two MNPs with anti-prostate cancer we reported in our previous studies. Elaiophylin showed potent antitumor activity against CRPC *in vitro* and *in vivo* by acting as a novel RORγ antagonist (Zheng et al., 2020). Ilicicolin A exerted antiproliferative activity in human PCa cells *via* inhibiting the EZH2 pathway; moreover, it could enhance the anticancer activity of enzalutamide in CRPC cancer models (Guo et al., 2021).

**FIGURE 1:**
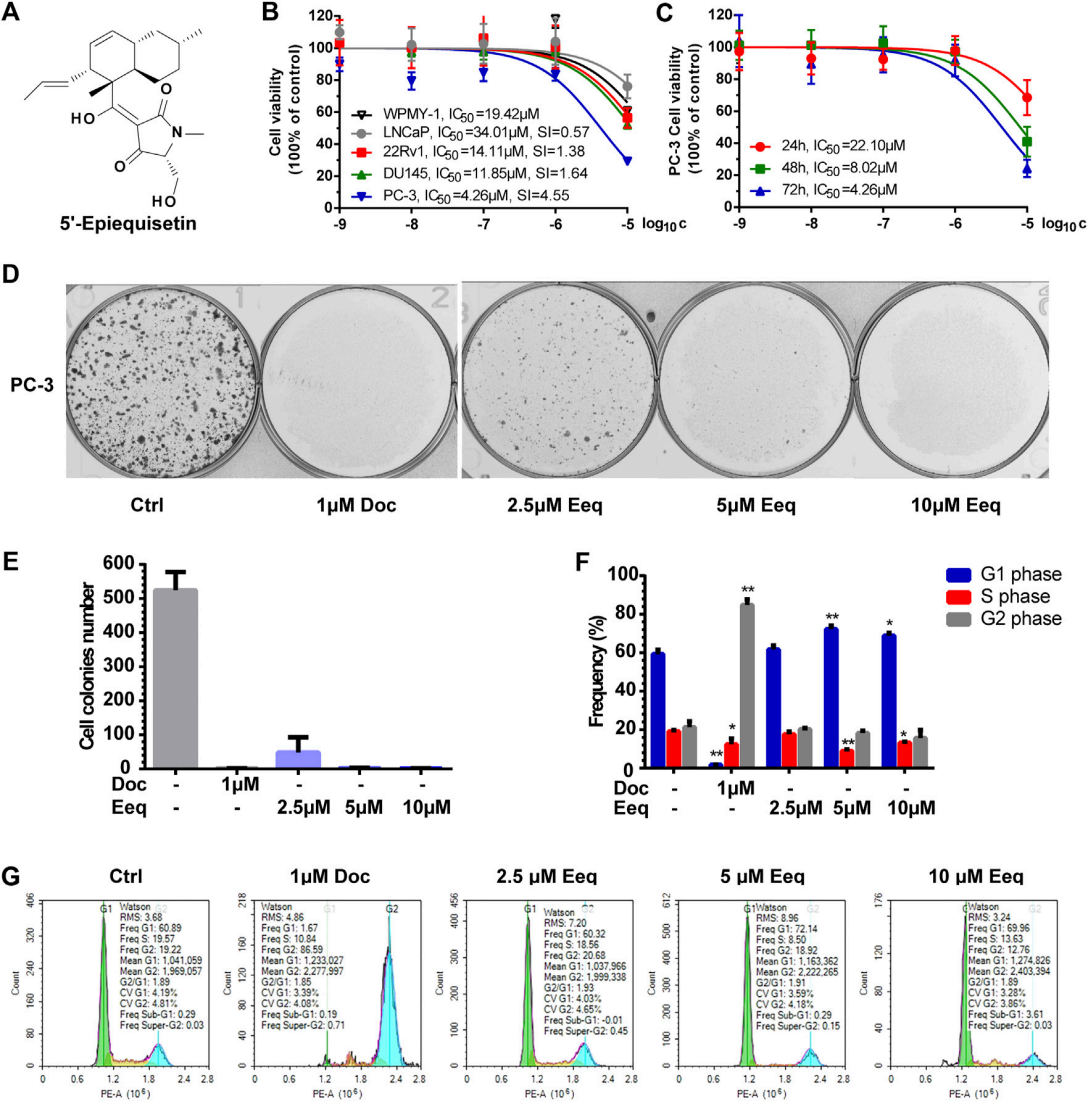
5′-Epiequisetin (Eeq) inhibited the proliferation of prostate cancer cells. **(A)** Chemical structure of Eeq. **(B)** Eeq inhibited prostate cancer cell viability to varying degrees. **(C)** Eeq inhibited PC-3 cells in a time-dependent manner. **(D,E)** Eeq reduced PC-3 cell colony formation. **(F,G)** Eeq altered the distribution of PC-3 cell cycle and arrested the cell cycle in the G1 phase. *p < 0.5, **p < 0.01 *vs*. control.

5′-Epiequisetin (Eeq) (Figure 1A) was identified as a tetramic acid antibiotic from a marine sponge-derived fungus, *Fusarium equiseti* SCSIO 41019, in our previous study (Chen et al., 2019). Relatively few studies have been reported on Eeq; however, more studies have been reported on its isomer equisetin, and it has been much studied for its antibacterial activity (Zhang et al., 2018; Chen et al., 2021; Zhang Q. et al., 2021). A recent study found that equisetin could produce anti-obesity effects by targeting 11 *β*-HSD1 (Xu et al., 2022). These studies suggest that this chemical structure has the potential for multiple biological activities. In screening experiments for anti-prostate cancer candidate compounds, we found that Eeq could inhibit the cell viability of four PCa cell lines (LNCaP, 22Rv1, DU145, and PC-3) to varying degrees, with the most dramatic effect on PC-3 cells. This finding inspired us to initiate a series of more in-depth studies on this compound in the field of anti-prostate cancer.

We first assessed its effects on PC-3 cell proliferation, apoptosis, and migration *in vitro*. Second, we sought to elucidate how it acts. Finally, we examined its anti-prostate cancer effects *in vivo* by establishing a xenograft tumor mouse model. Our work provided evidence for an MNP possessing an anti-PCa effect both *in vitro* and *in vivo*, suggesting a potential anti-PCa agent. More immediately, our results should aid researchers interested in the pursuit of compounds with similar structures for the development of anti-PCa drugs, identifying candidate molecules that have potential for further development. In this way, we hope this work serves in part as a catalyst to move towards the production of more robust anti-PCa molecules.

## Materials and methods

### Reagents and antibodies

Docetaxel was purchased from Selleck Chemicals (Cat# RP56976). RPMI 1640 medium was purchased from Gibco (Cat# 8120018). Fetal bovine serum was purchased from Gemini (Cat# 900-108). Charcoal stripped FBS was purchased from Biological Industries (Cat# 04-201-1A). Thiazolyl blue tetrazolium bromide (Cat# M8180) and crystal violet stain solution (Cat# G1063) were purchased from Solarbio life sciences. Human apoptosis array kit was from R&D Systems (Cat# ARY009). NuPAGETM 10% Bis-Tris Gel (Cat# NP0302BOX), eBioscience™ Annexin V-FITC apoptosis detection kit (Cat# BMS500FI-300), FxCycle™ PI/RNase staining solution (Cat# F10797) and Vybrant cell-labeling solutions (V22885) were purchased from Invitrogen. All antibodies used in this study were purchased from Proteintech Group (CDK4, Cat# 66950-1-lg; CDK6, Cat# 66278-1-lg; Cyclin D1, Cat# 26939-1-AP; RB1, Cat# 10048-1-lg; E2F1, Cat# 66515-1-lg; Survivin, Cat# 66495-1-lg; p21, Cat# 10355-1-AP). Eeq was isolated and identified from the marine sponge-derived fungus *Fusarium equiseti* SCSIO 41019 by various chromatographic and comprehensive spectroscopic methods in our previous study (Chen et al., 2019), respectively. It was determined to have ≥95% purity by analytical HPLC.

### Cell culture

LNCaP, 22Rv1, DU145, PC-3, and WPMY-1 cells were obtained from the National Collection of Authenticated Cell Cultures (Shanghai, China). All cell lines were identified by STR, and the cells used in the experiment were within 15 passages. LNCaP and 22Rv1 cells were maintained in RPMI 1640 supplemented with 10% (v/v) fetal bovine serum (FBS), 100 units/mL penicillin, and 100 μg/μL streptomycin; DU145 cells were cultured in MEM plus with the same additives; PC-3 cells were cultured in DMEM F12 plus with the same additives; WPMY-1 cell line was maintained in DMEM (Gibco, China) supplemented with 5% (v/v) fetal bovine serum (FBS) (Biological Industries, Israel), 100 units/mL penicillin, and 100 μg/μL streptomycin.

### MTT assay

Cell viability was analyzed using MTT assay as previously described (Wang et al., 2021). In brief, cells were seeded overnight in a 96-well plate at a density of 5*10^3^ per well and then treated with a range of concentrations of Eeq for the intended time. After the cells were exposed to Eeq for a certain time, the MTT solution was added to the cell culture wells for 4 h. Then, OD_570_ values were detected by using a Hybrid Multi-Mode Reader (Synergy H1, BioTek). The experiment was repeated three times independently. IC_50_ was calculated by using GraphPad 9.0 software. The degree of selective cytotoxicity index was expressed as SI = IC_50_ in normal cells/IC_50_ in tumor cells.

### Plate clone formation assay

PC-3 cells were seeded in a 6-well plate at a density of 1000 per well overnight, and then they were treated with DMSO (0.1%, v/v) and docetaxel (1 μM), Eeq (2.5, 5, 10 μM) for demanded time, respectively. The culture medium was changed every 72 h until the obvious cell cloning colonies were formed about 15 days later, and then the cells were fixed with 4% formaldehyde for 30 min, discarded 4% formaldehyde and washed cells with PBS buffer, and stained the colonies with crystal violet stain solution. After the cells had been stained for 30 min, we discarded the staining solution, washed the cells with PBS buffer, and then took colonies’ photos with a colony counter (GelCount, Oxford Optronix). The experiment was repeated three times independently.

### Apoptosis and cell cycle assay

PC-3 cells were seeded in a 6-well plate at a density of 2.0*10^5^ per well overnight and treated with DMSO (0.1%, v/v), docetaxel (1 μM), Eeq (2.5, 5, and 10 μM), respectively, for 48 h. The cells were collected and stained according to the instructions of eBioscience™ Annexin V-FITC apoptosis detection kit and FxCycle™ PI/RNase staining solution, respectively. Then, cell apoptosis and cell cycle distribution were detected using flow cytometry (NovoCyte, Agilent). This experiment was repeated three times independently.

### Cell migration assay

A gradient concentration of Eeq (1.25, 2.5, 5, 10, 20, and 40 μM) solution was prepared with 10% FBS DMEM F12 medium in a 96 well U-shaped plate, and then these solutions were transferred to the lower chamber in CIM-plate 16 (5665817001, Agilent) with 2 wells for each concentration. Next, the upper chamber of the CIM-plate 16 was mounted directly above the lower chamber to form a complete plate. PC-3 cells were seeded in the upper chamber of the CIM-plate 16 at a density of 2.5*10^5^. Eeq was then added to the upper chamber so that the concentration of Eeq matched that of the lower chamber. After that, the CIM-plate 16 was installed on a real-time cell analyzer (xCELLigence RTCA DP Instrument, Agilent) to detect the effect of Eeq on the migration of PC-3 cells.

### Human apoptosis array

PC-3 cells were seeded in T75 Flask at a density of 5.0*10^6^ for 48 h and treated with DMSO (0.1%, v/v) and Eeq (10 μM) for 48 h, respectively. Then, cells were collected, and proteins were extracted and analyzed according to the instructions of the human apoptosis array kit.

### qPCR detection of endogenous gene expression

qPCR was performed as described previously (Wang et al., 2021). In brief, PC3 cells were seeded into 6-well plates and grown in DMEM F12 medium containing 10% FBS. After attachment, cells were treated with Eeq for 24 h. Then RNA was extracted and purified using the TRIzol reagent (15596018, Life technologies). cDNA was prepared from 1 μg of RNA with RevertAid Master Mix (M1632, Thermo Fisher Scientific). Diluted cDNA was used to perform quantitative RT-PCR (LightCycler96, Roche) using PowerUp SYBR Green master mix (A25742, applied biosystems) with GAPDH as the internal standard. Primers for quantitative RT-PCR were listed in Table 1. Experiments were performed in triplicate.

**TABLE 1:** List of primer sequences for quantitative PCR.

Primer code	Primer sequence (5′-3′)	Product length
DR5 forward	GGA​TGG​TCA​AGG​TCG​GTG​ATT​GTA​C	146 bp
DR5 reverse	GAG​AGA​ACA​GGG​AGA​GGC​AGG​AG	
GAPDH forward	CAG​GAG​GCA​TTG​CTG​ATG​AT	138 bp
GAPDH reverse	GAAGGCTGGGGCTCATTT	

### Western blot

PC-3 cells were seeded in 60 mm dishes at a density of 1.0*10^6^ for 48 h. Then, cells received a fresh medium containing the indicated compounds, docetaxel (1 μM), and the indicated concentrations of Eeq (2.5, 5, and 10 μM). The whole cell extracts were prepared after 48 h by using the mixture of RIPA (R0020, Solarbio life science) and PMSF (P0100, Solarbio life science). Proteins were analyzed on 10% SDS-PAGE gel sand transferred to nitrocellulose membranes. In this study, 30 μg of total protein was used to detect all proteins except DR5, which was measured by using 60–80 μg of total protein. All information of antibodies is listed in the section on reagents and antibodies. Images were captured using the Chemidoc CD Touch (Bio-Rad, United States), and images were analyzed and processed using the Image Lab 6.0 (Bio-Rad, Chinese edition).

### Small interfering RNA silence DR5 expression

PC-3 cells were seeded in a 6-well plate at a density of 2.0*10^5^ for 48 h, and then cells were transfected with si-RNA (small interfering RNA sequences are shown in Table 2) by using Lipofectamine 2000 (11668019, Thermo Fisher Scientific) according to the manufacturer’s manual. After 48 h of transfection, Eeq was added to the 6-well plate, and the cells were exposed to the compound for another 48 h. Then, apoptosis was detected using flow cytometry.

**TABLE 2:** List of small interfering RNA sequences for silencing DR5.

Primer code	Primer sequences (5′-3′)
hDR5-674 (sense)	CCA​CAA​AGA​AUC​AGG​UAC​AAA​TT
hDR5-674 (antisense)	UUU​GUA​CCU​GAU​UCU​UUG​UGG​TT
hDR5-451 (sense)	CUC​ACU​GGA​AUG​ACC​UCC​UUU​TT
hDR5-451 (antisense)	AAA​GGA​GGU​CAU​UCC​AGU​GAG​TT
hDR5-1382 (sense)	GCA​GAA​GAU​UGA​GGA​CCA​CUU​TT
hDR5-1382 (antisense)	AAG​UGG​UCC​UCA​AUC​UUC​UGC​TT
Negative control (sense)	UUC​UCC​GAA​CGU​GUC​ACG​UTT
Negative control (antisense)	ACG​UGA​CAC​GUU​CGG​AGA​ATT

### Xenograft tumor model

Animal experiments were performed according to procedures approved by the Guangxi University of Chinese Medicine Institutional Animal Ethical and Welfare Committee (Approval code: 20210603-071). Male BALB/c nude mice (Hunan SJA laboratory animal Co., Ltd., China) of 5–6 weeks old were acclimated to a sterilized normal diet and water for seven days. Mice were inoculated subcutaneously with 5*10^5^ androgen-independent PC-3 human prostate cancer cells. The PC-3 cell line was purchased from Cell Bank/Stem Cell Bank, The Committee of Type Culture Collection of the Chinese Academy of Sciences (Shanghai, China), where the cells were authenticated by STR DNA profiling and tested as *mycoplasma* free. The intervention started when tumors reached a volume of 50–100 mm^3^ about one week after inoculation. Mice were randomly assigned into three groups (*n* = 6-7): vehicle, 10 mg/kg docetaxel, 20 mg/kg, and 40 mg/kg Eeq. Docetaxel and Eeq were dissolved in a solvent containing 5%DMSO, 30% PEG300, 5% Tween 80 and dd H_2_O. Docetaxel was intraperitoneally administered twice a week for four weeks, and Eeq was administered once a day throughout the study. Tumor size was measured twice a week with calipers. Tumor volume was calculated by using the formula: π/6*length*width*width (Wang et al., 2016). Mouse body weight was measured twice a week. Mice were sacrificed when they had received 4-week intervention treatment in observance of the institutional guideline on tumor size. Xenograft tumors were harvested, weighed, and fixed with 4% paraformaldehyde and/or snap frozen and stored in liquid nitrogen.

### Statistical analysis

All analyses were conducted using GraphPad Prism 9 (GraphPad Software, United States). All results were presented as mean ± standard deviation (SD) and analyzed using one-way ANOVA. Comparisons between the groups were conducted using Dunnett’s *t*-test. Level for statistical significance was set at *p < 0.05, **p < 0.01.

## Results

### 5′-epiequisetin suppressed prostate cancer cells proliferation *via* inhibiting the PI3K/Akt signaling pathway

Eeq inhibited the cell viability of prostate cancer cells (LNCaP, 22Rv1, DU145, and PC-3) and normal prostatic cells (WPMY-1) in varying degrees in a dose-dependent manner by MTT assay, among which, it showed the most significant effect on PC-3 cells (IC_50_ = 4.43 ± 0.24 μM). The selective cytotoxicity index (SI) of Eeq for PC-3, DU145, 22Rv1 and LNCaP cells were 4.55, 1.64, 1.38, and 0.57, respectively, suggesting that Eeq has the best selectivity for PC-3 cells (Figure 1B). Then, we used the MTT assay to further investigate the effect of Eeq on the viability of PC-3 cells. We found that the cytotoxicity of Eeq on PC-3 cells was time-dependent (Figure 1C). Next, we performed a plate clone formation assay to investigate the effect of Eeq on the proliferation of PC-3 cells. The results showed that it could significantly inhibit the clone formation of PC-3 cells in a dose-dependent manner (Figures 1D and E). Notably, the PC-3 cell line is a prostate cancer cell that does not express the androgen receptor, and these findings suggest that Eeq does not exert its anti-prostate cancer effects through modulation of the androgen receptor signaling pathway. It must then exert its effects through other pathways.

In order to reveal how it inhibited the proliferation of prostate cancer cells, we detected the cell cycle distribution of PC-3 cells. As shown in Figures 1F and G, when being treated with docetaxel, which is an M-phase blocker, a large number of PC-3 cells were arrested at the M phase, and the proportion of G2/M phase cells increased sharply. However, unlike in the case of docetaxel, the percentage of the G1 phase increased significantly in the PC-3 cells which were treated with Eeq. These findings point to the fact that Eeq blocks the cell cycle at the G1 phase, resulting in the inability of cells to proliferate.

**FIGURE 2:**
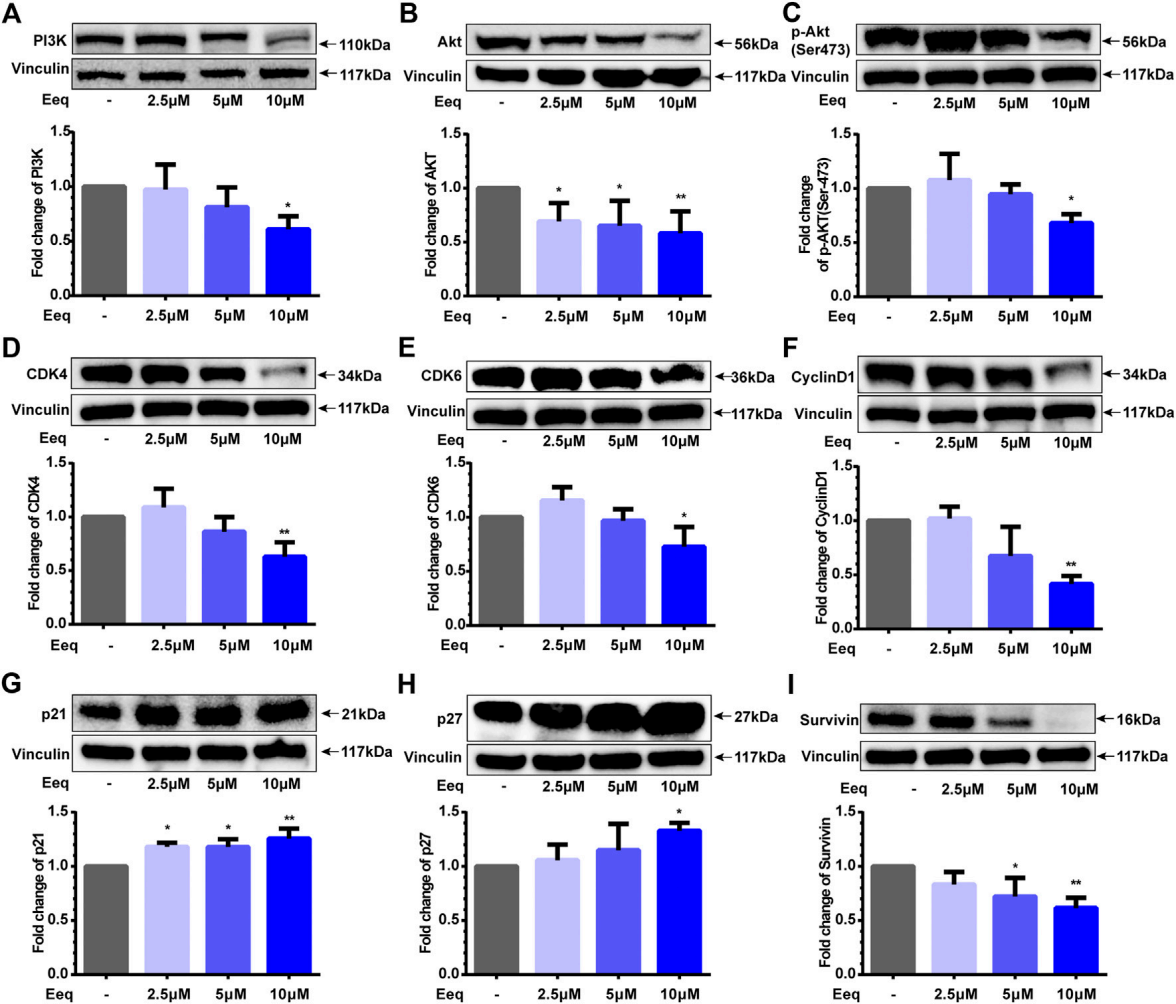
5′-Epiequisetin (Eeq) regulated the expression of proliferation-related proteins. **(A)** PI3K, **(B)** Akt, **(C)** phosphorylated Akt, **(D)** CDK4, **(E)** CDK6, **(F)** cyclin D1, **(G)** p21, **(H)** p27, and **(I)** survivin. *p < 0.5, **p < 0.01 *vs*. control.

Cyclin D, CDK4, and CDK6 are fundamental drivers of the cell cycle in driving G1 to S phase transition (Goel et al., 2017). In order to clarify the mechanism that Eeq blocked the cell cycle in the G1 phase, we performed Western blot to detect the expression of the proteins above. As shown in Figure 2, Eeq did reduce the expression of Cyclin D, CDK4, and CDK6 proteins while upregulating the expression of p21 and p27 proteins (Figures 2D–H), which are also key roles in regulating the cell cycle from G1 phase to S phase (Coqueret, 2003). Furthermore, PI3K/Akt/p21 signaling axis is a key pathway to regulate the cell cycle (Chang et al., 2003). In the present study, Eeq inhibited PI3K and Akt protein expression by reducing Akt phosphorylation at Ser473 (Figures 2A–C). Finally, it declined the expression of survivin protein, leading to cell death (Figure 2I). Based on the findings above, it can be concluded that Eeq inhibited the function of the PI3K/Akt/p21 signaling pathway, which, in turn, blocked the G1 phase of the cell cycle, resulting in the inability of cells to divide and the eventual loss of proliferative capacity.

### 5′-epiequisetin restrained prostate cancer cells migration *via* suppressing the beta-catenin/cadherin signaling pathway

**FIGURE 3:**
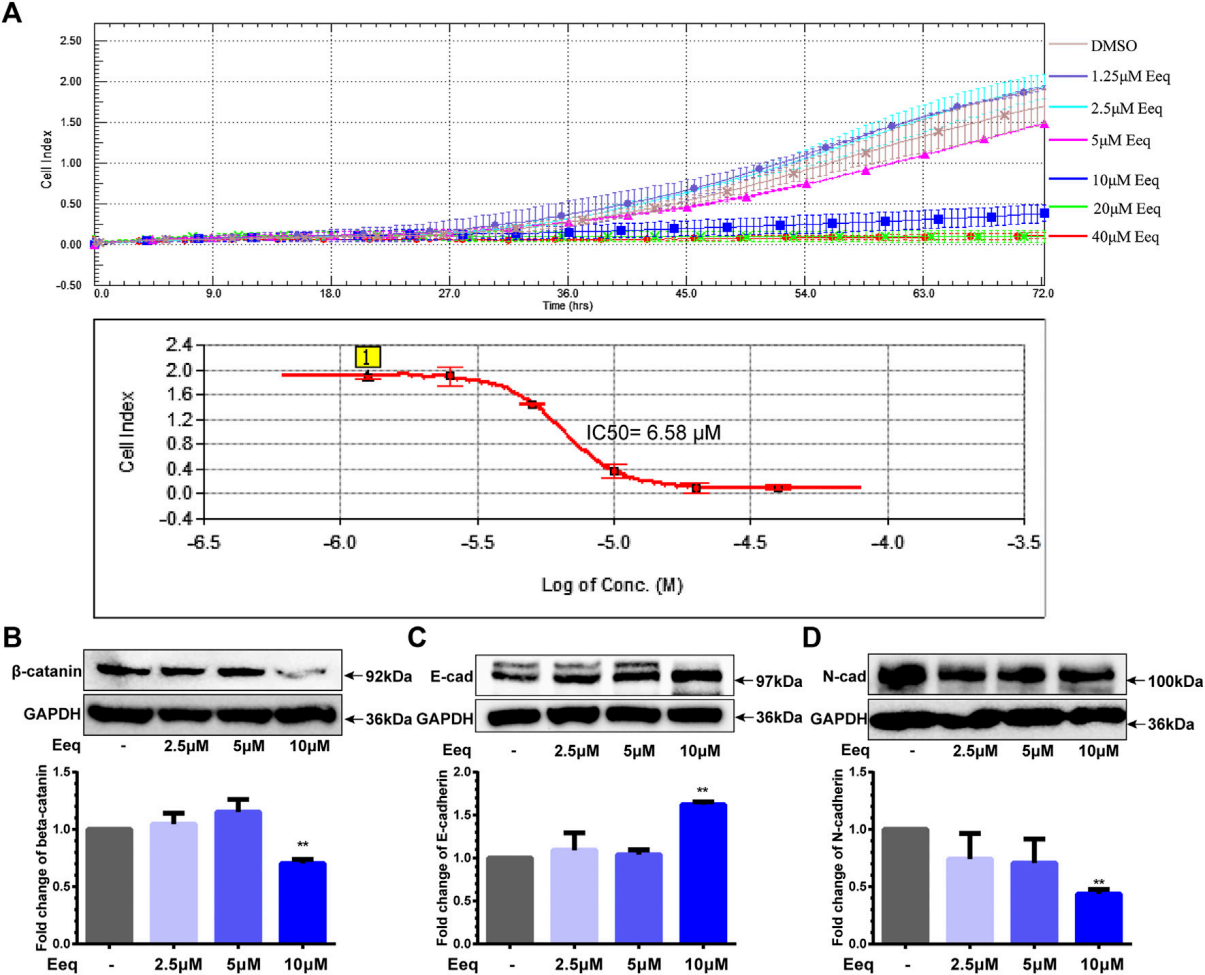
5′-Epiequisetin (Eeq) inhibited the migration of PC-3 cells. **(A)** IC_50_ of Eeq inhibited PC-3 cell migration. **(B)** Impact of Eeq on protein expression of beta-catenin. **(C)** Impact of Eeq on protein expression of E-cadherin. **(D)** Impact of Eeq on protein expression of N-Cadherin. *p < 0.5, **p < 0.01 vs. control.

On the other hand, we found that Eeq inhibited PC-3 cell migration with an IC_50_ value of 6.80 ± 0.31 μM by real-time cell analysis (Figure 3A). In addition to directly corresponding to the cell cycle, the PI3K/Akt signaling pathway also plays a key role in cell invasion and migration (Hamidi et al., 2017). It is well known that beta-catenin is involved in WNT and/or PI3K/Akt signaling pathways which regulate cancer cell migration, thereby it is considered a target for the treatment of cancer (Tang et al., 2019; Zhang et al., 2020). We found that Eeq decreased the protein expression of *β*-catenin and N-cadherin and increased the expression of E-cadherin (Figures 3B–D), while it showed no impact on WNT5A/5B (Supplementary Figure S2F). These findings may provide evidence that Eeq attenuated epithelial–mesenchymal transitions through PI3K/Akt/beta-catenin/cadherin in prostate cancer cells, thereby inhibiting the migration of PC-3 cells.

### 5′-epiequisetin induced PC-3 cells apoptosis through the DR5 signaling pathway

**FIGURE 4:**
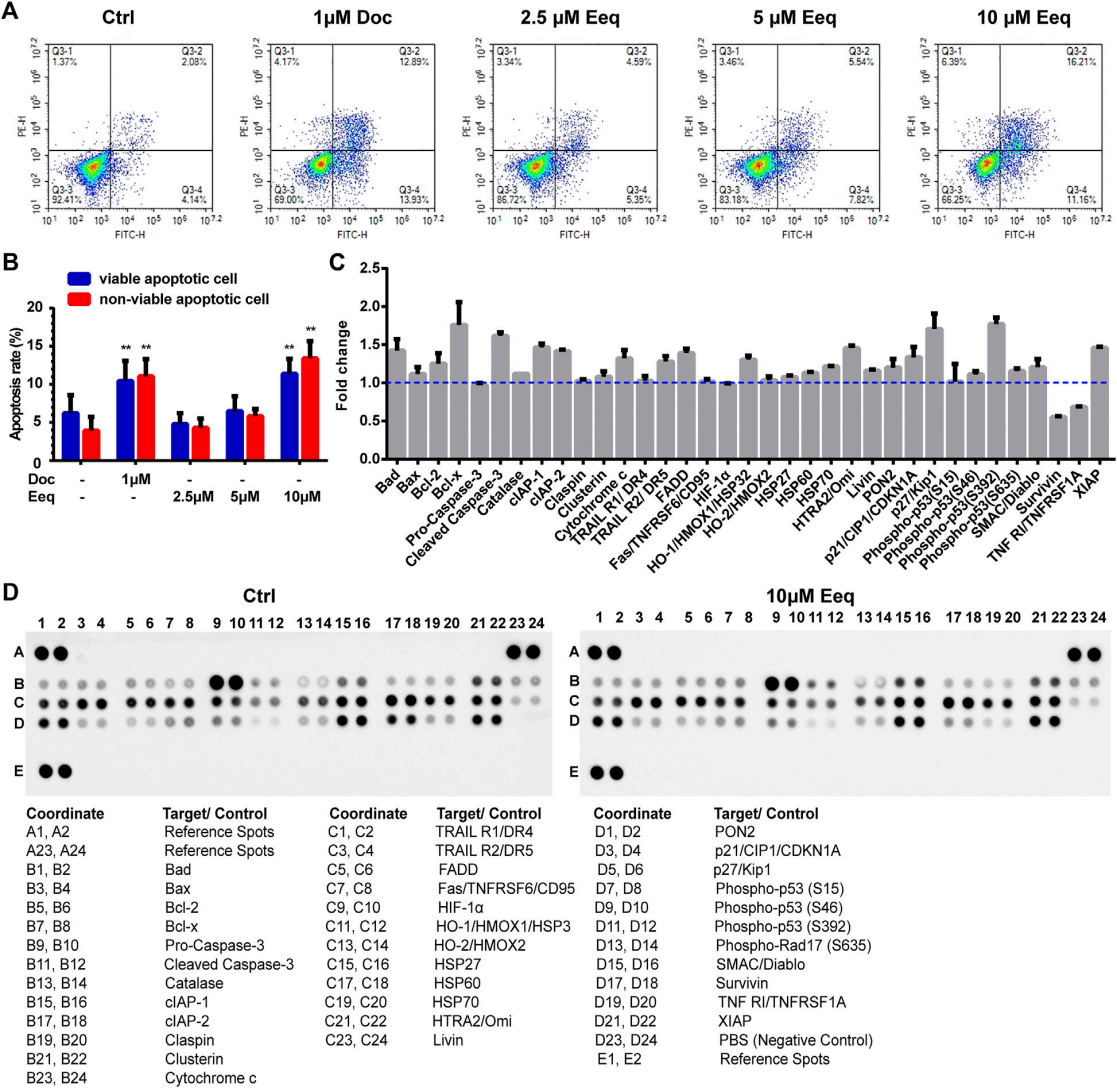
5′-Epiequisetin (Eeq) induced PC-3 cell apoptosis. **(A,B)** Eeq induced PC-3 cell apoptosis in a dose-dependent manner. **(C,D)** Eeq regulated the expression of apoptosis-regulated proteins. *p < 0.5, **p < 0.01 vs. control.

To further identify how Eeq produced cytotoxicity, we used flow cytometry to detect the apoptosis of PC-3 cells. As shown in Figures 4A and B, Eeq induced cell apoptosis in a dose-dependent manner with docetaxel acting as a positive control. In order to clarify how Eeq induced apoptosis, we performed a human apoptosis array. As shown in Figures 4C and D, Bcl-x, cleaved caspase-3, DR5, p21, and p27, were upregulated after Eeq had been treated for 48 h. However, the survivin protein that promotes cell survival was downregulated. Among the apoptosis proteins encapsulated in the human apoptosis array, DR4, DR5, TNFRSF6, and TNFRSF1A are proteins involved in the TNF signal transduction pathway, which is an important pathway that mediates apoptosis. DR5 was upregulated after Eeq had been treated for 48 h, while DR4, TNFRSF6, and TNFRSF1A were not upregulated by Eeq (Figure 4C). Therefore, we studied DR5 in the follow-up work. We noticed that DR5 was a cell membrane receptor that mediated cell apoptosis. These findings inspired us to speculate that Eeq mediated apoptosis by driving the DR5 signaling pathway. Then, in order to confirm this hypothesis, we conducted a series of experiments.

**FIGURE 5:**
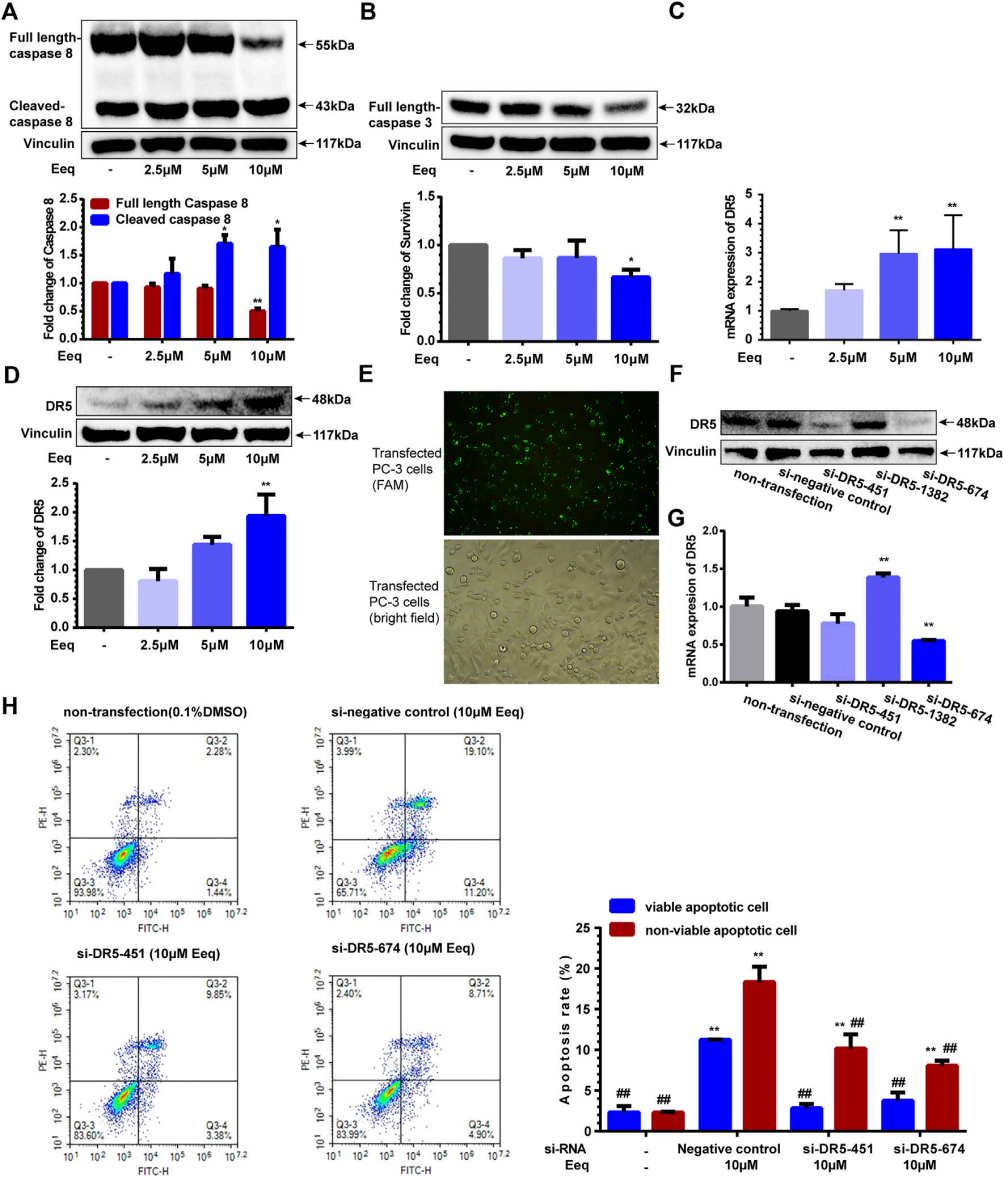
5′-Epiequisetin (Eeq) induced PC-3 cell apoptosis by activating the DR5 signaling pathway. **(A)** Eeq regulated caspase 8 protein expression. **(B)** Eeq reduced caspase 3 protein expression. **(C)** Eeq increased mRNA expression of DR5. **(D)** Eeq increased protein expression of DR5. **(E)** PC-3 cells were transfected with small interfering RNA. **(F)** Small interfering RNA knocked down DR5 protein expression. **(G)** Small interfering RNA knocked down DR5 mRNA expression. **(H)** Eeq induced a decrease in apoptosis of PC-3 cells after DR5 was knocked down by small interfering RNA. *p < 0.5, **p < 0.01 *vs*. control; ^#^ *p* < 0.5, ^##^ *p* < 0.01 *vs*. negative control (si-RNA).

Activation of caspase-8 and caspase-3 is the key link in the induction of apoptosis by DR5 (Muñoz-Pinedo and López-Rivas, 2018). In this study, caspase-8 and caspase-3 were activated, as evidenced by a decrease in the full length of caspase-8/3 and an increase in cleaved caspase-8 (Figures 5A and B). Furthermore, we found Eeq increased expression of DR5 on both mRNA and protein levels (Figures 5C and D). Most importantly, the apoptotic cells induced by Eeq were significantly reduced (Figure 5H) when DR5 expression was silenced by small interfering RNA (Figures 5E–G). Taken together, Eeq induced PC-3 cell apoptosis through the DR5 signaling pathway.

### Tables 5′-Epiequisetin suppressed the growth of prostate cancer *in vivo*

**FIGURE 6:**
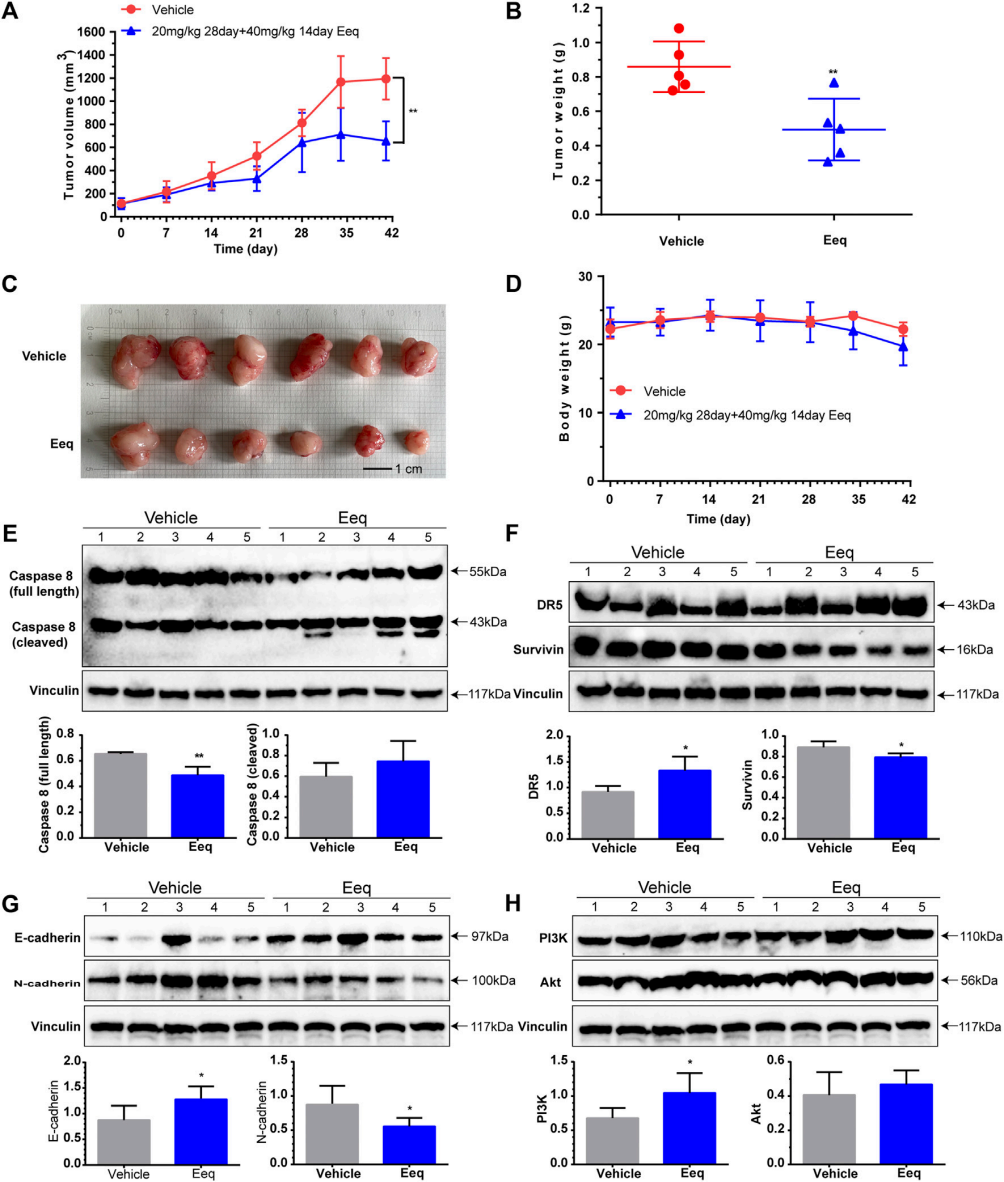
5′-Epiequisetin (Eeq) suppressed prostate cancer development *in vivo*. **(A)** Eeq reduced tumor volume in mice. **(B)** Eeq decreased tumor weight in mice. **(C)** Tumor size and appearance in mice. **(D)** Body weight of mice during administration. **(E)** Eeq regulated the expression of caspase 8 protein in mice tumor tissue. **(F)** Eeq increased DR5 protein and decreased survivin protein expression in mice tumor tissue. **(G)** Eeq increased E-cadherin protein and decreased N-cadherin protein expression in mice tumor tissue. **(H)** Eeq had no significant effect on PI3K and Akt protein expression in tumor tissue of mice. *p < 0.5, **p < 0.01 *vs*. vehicle.

In order to further evaluate the anti-PCa effect of Eeq *in vivo*, we constructed a PCa xenograft model by subcutaneously injecting PC-3 cells into the right flank of the mice. In the first experiment, we divided the model mice into vehicle group, docetaxel group (10 mg/kg), and Eeq group (10–20 mg/kg). The Eeq concentrations tested in mice refer to one of our previous studies. Ilicicolin A is also a marine compound which was isolated from the Beibu Gulf coral–derived fungus *Acremonium sclerotigenum* GXIMD 02501. It showed antiproliferative activity in human prostate cancer at 2.5–10 μM *in vitro* and at 10 mg/kg *in vivo* (Guo et al., 2021). The inhibitory activity of Eeq on prostate cancer proliferation *in vitro* is similar to that of ilicicolin A, so we set the 10 mg/kg dose as the initial dose of Eeq *in vivo*. We found that Eeq showed tumor growth inhibition in only half the number of mice at a dose of 10–20 mg/kg. In general, the tumor growth inhibition of 10–20 mg/kg Eeq was less effective than that of 10 mg/kg docetaxel in terms of tumor size and weight (Supplementary Figure S1). Then, we carried out the second experiment and changed the dose of Eeq to 20–40 mg/kg. Herein, 20–40 mg/kg Eeq dramatically inhibited tumor growth in mice, evidenced by a sharp decline in tumor size and weight (Figures 6A–C). Moreover, all mice showed good tolerance to 20–40 mg/kg Eeq, and no mice died during the experiment. There were no significant changes in body weight and organ index (Figure 6D; Supplementary Figures S2A–E). Furthermore, we also examined mice tumor tissues and found that Eeq also promoted DR5 and E-cadherin while inhibiting expression of caspase 8 (full length), surviving, and N-cadherin *in vivo* (Figures 6E–G). However, the expression of PI3K and Akt proteins in mice tumor tissue was not inhibited as *in vitro* (Figure 6H), suggesting that Eeq could not affect this signaling pathway in mice. Based on the findings above, we confirm that Eeq played an anti-PCa role through DR5 and beta-catenin/E-cadherin signaling pathways.

## Discussion

PI3K/Akt signaling pathway is involved in the regulation of cell survival, cell cycle progression, and cellular growth, which has been widely recognized. It was found to be commonly activated in human cancer, thereby suppressing the function of this signaling pathway has become a strategy for treating cancer (Alzahrani, 2019). In the present study, we found that Eeq induced cell cycle to be arrested at the G1 phase terminated cell division, resulting in inhibition of PCa cell proliferation. We traced the PI3K/Akt signaling pathway that regulates the cell cycle, and we found that Eeq reduced PI3K and Akt protein expression and inhibited Akt phosphorylation at Ser473 *in vitro*. These findings evidenced that Eeq inhibited cell proliferation through negative regulation of PI3K/Akt signaling in PC-3 cells *in vitro*. However, assays on mice tumor tissue showed that Eeq did not reduce the expression of PI3K and Akt proteins as it did at the cellular level. These data showed that the results of PI3K and Akt proteins *in vitro* and *in vivo* are not consistent. These findings suggested that the role of Eeq on PI3K and Akt protein at the cellular level may not be shown at the integrated animal level.

Epithelial–mesenchymal transition (EMT) was found to enhance tumor cell metastasis, chemoresistance, and tumor stemness (Dongre and Weinberg, 2019; Lu and Kang, 2019). The increased expression of N-cadherin and the decreased expression of E-cadherin were associated with the development of EMT (Loh et al., 2019). Drug therapies that target these proteins have thus been developed (Mariotti et al., 2007; Wong et al., 2018). In this study, we found Eeq decreased expression of N-cadherin and increased expression of E-cadherin. This finding indicated that Eeq might alleviate and/or reverse the EMT process. The EMT process is regulated by a complex network of signaling pathways and transcription factors, including the PI3K/Akt/beta-catenin and Wnt/beta-catenin signaling pathways (Gonzalez and Medici, 2014). It is well known that the interaction between E-cadherin and *β*-catenin occurs during the EMT process (Schmalhofer et al., 2009), so we tracked the protein expression of beta-catenin and Wnt. We found Eeq indeed down-regulated the beta-catenin while it showed no impact on Wnt protein expression. Furthermore, we detected E-cadherin and N-Cadherin in mice tumor tissue and obtained consistent results at the cellular level. Conclusively, Eeq inhibited cell migration by attenuating the EMT process, which was related to the beta-catenin/cadherins signaling pathway.

DR5 is a member of the tumor necrosis factor receptor superfamily, which can initiate the apoptosis pathway by binding to its associated ligand, including synthetic and natural agents (Yuan et al., 2018). Therefore, it is considered to be a cancer treatment strategy. Bioymifi was the first DR5 activator reported in 2013, and it could bind directly to DR5 to activate the caspase 8-induced apoptosis signaling pathway, leading to human glioblastoma (T98G) cells apoptosis (Wang et al., 2013). In the present study, we found that Eeq also activated the caspase 8-induced apoptosis signaling pathway *in vitro*. We further confirmed that Eeq increased DR5 expression at both mRNA and protein levels. More intriguingly, when DR5 was silenced by small interfering RNAs, Eeq-induced apoptosis was drastically reduced. We also detected DR5 and Caspase 8 protein expression in mice tumor tissue and obtained consistent results at the cellular level.

These data suggest that Eeq induced apoptosis through DR5 both *in vivo* and *in vitro*, thus, exerting anti-PCa effects. To our knowledge, there are few reports on the biological activity of Eeq except the antibacterial effects hitherto. In this study, we first proved its anti-PCa effect from three phenotypes of cell proliferation, migration, and apoptosis and then revealed how it produced these effects. According to our current data, we concluded that Eeq could play anti-PCa roles through PI3K/Akt and DR5 signaling pathways. We also realize that these findings may not be the whole picture of the anticancer effect of Eeq due to the limitations of detection methods. However, we confirmed that Eeq indeed had an anti-PCa effect *in vitro* and *in vivo*. In addition, it may also have effects to overcome other tumors with aberrant PI3K/Akt and DR5 signaling pathways. We believe that the present study shed the first light on the anti-prostate cancer capacity of Eeq. However, whether Eeq can be developed into an agent for the treatment of prostate cancer and other tumors needs more extensive and in-depth research.

## Data Availability

The original contributions presented in the study are included in the article/Supplementary Material; further inquiries can be directed to the corresponding author.
