# Supplementary material for: Analysis of regulating activities of 5′-epiequisetin on proliferation, apoptosis, and migration of prostate cancer cells in vitro and in vivo
Source: Front Pharmacol. 2022 Aug 10;13:920554. doi: 10.3389/fphar.2022.920554 (PMC9399367; doi:10.3389/fphar.2022.920554)
Supplement: Supplementary file 1 [file DataSheet1.docx]

Supplementary Material

# Supplementary Data

## Fig.2A in our manuscript represents the analysis outcomes of the whole uncropped images in Sup.M.Fig.1.


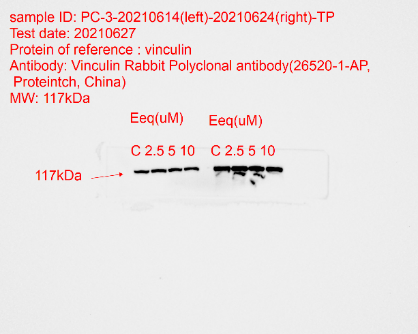

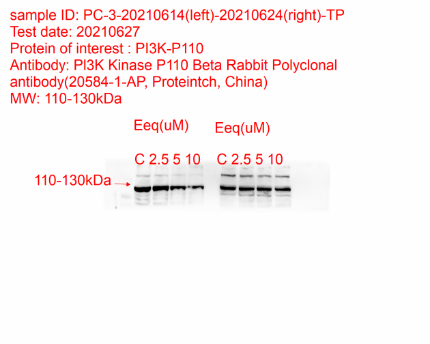


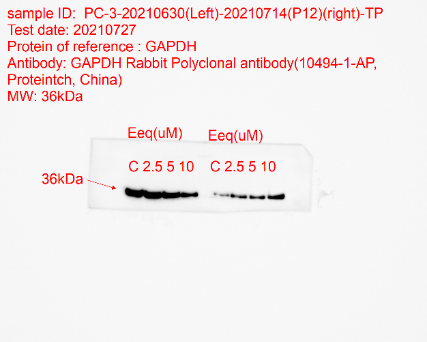

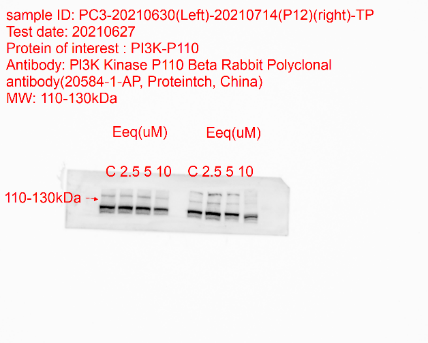


Sup.M.Fig.1 Original western blots images of PI3K.

## Fig.2B in our manuscript represents the analysis outcomes of the whole uncropped images in Sup.M.Fig.2.


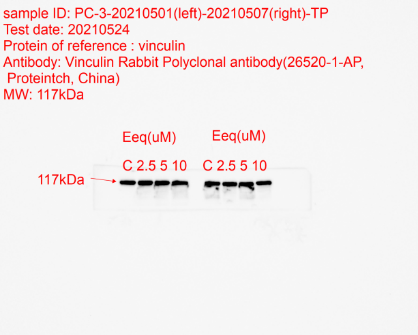

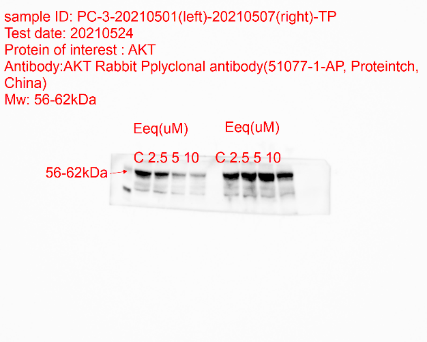


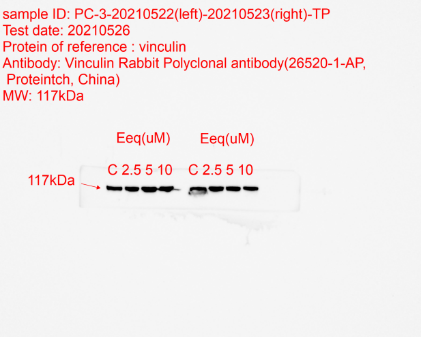

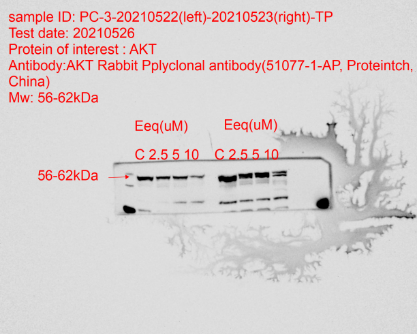


Sup.M.Fig.2 Original western blots images of Akt.

## Fig.2C in our manuscript represents the analysis outcomes of the whole uncropped images in Sup.M.Fig.3.


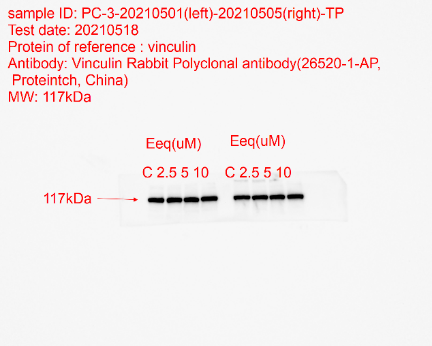

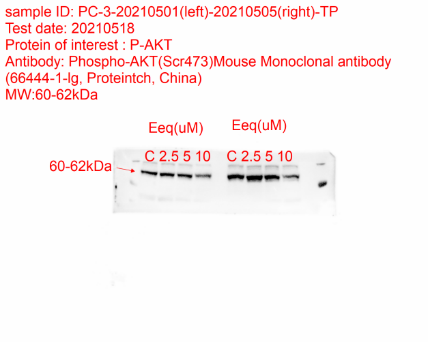


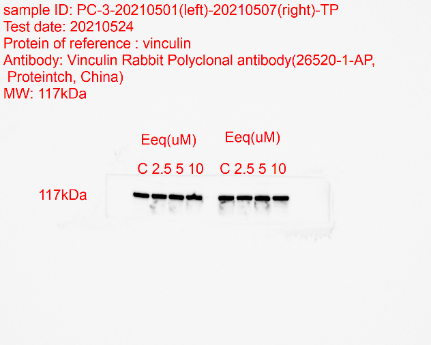

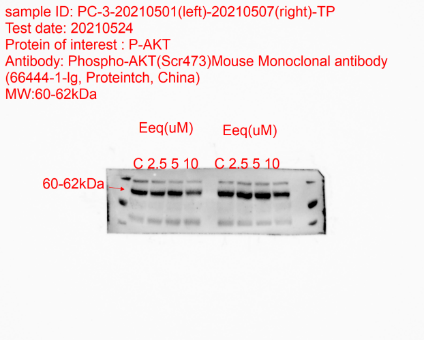


Sup. M.Fig.3 Original western blots images of p-Akt (Ser-473).

## Fig.2D in our manuscript represents the analysis outcomes of the whole uncropped images in Sup.M.Fig.4.


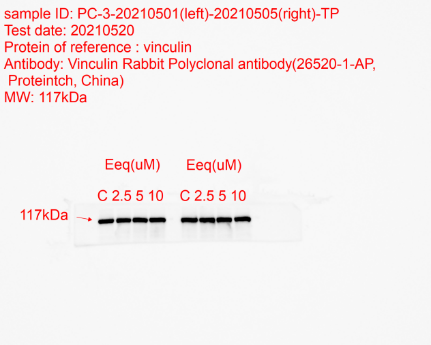

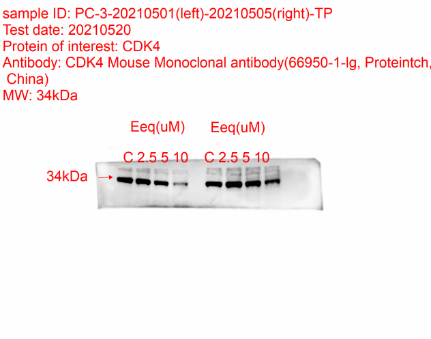


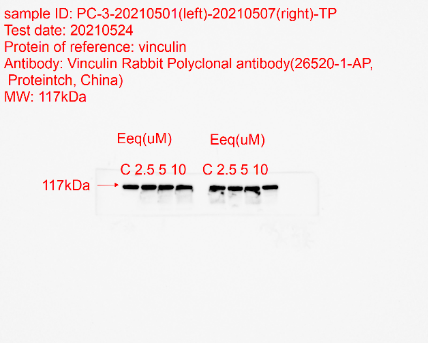

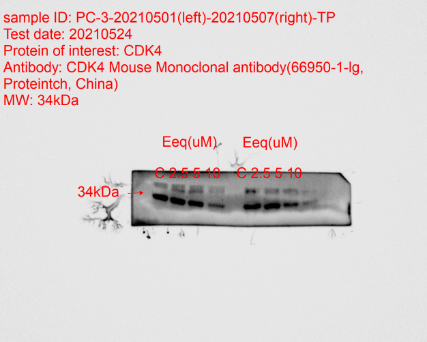


Sup. M.Fig.4 Original western blots images of CDK4.

## Fig.2E in our manuscript represents the analysis outcomes of the whole uncropped images in Sup.M.Fig.5.


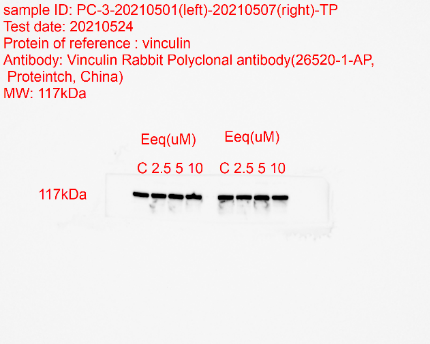

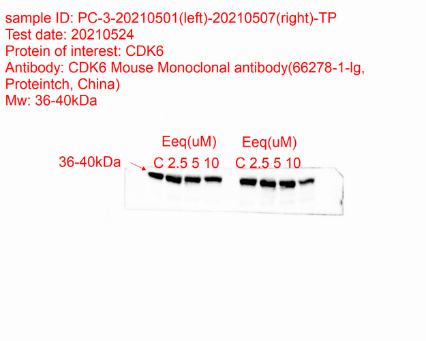


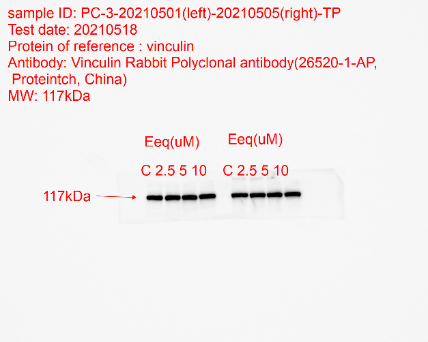

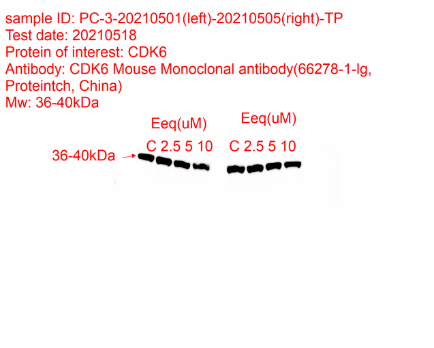


Sup. M.Fig.5 Original western blots images of CDK6.

## Fig.2F in our manuscript represents the analysis outcomes of the whole uncropped images in Sup.M.Fig.6.


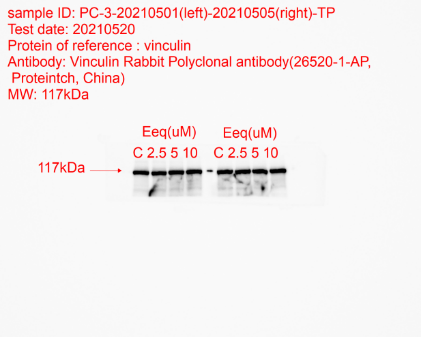

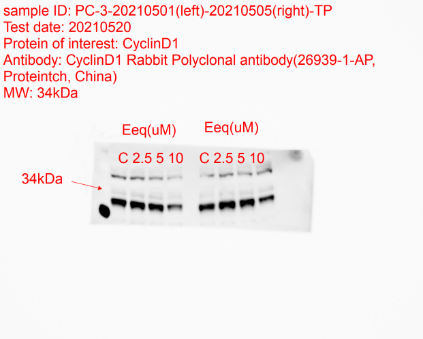


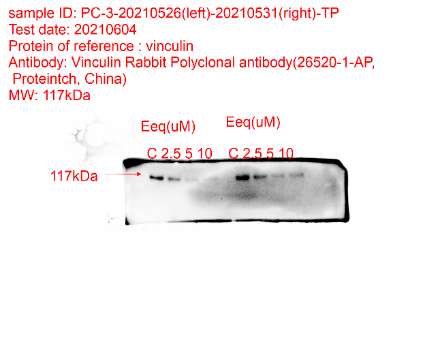

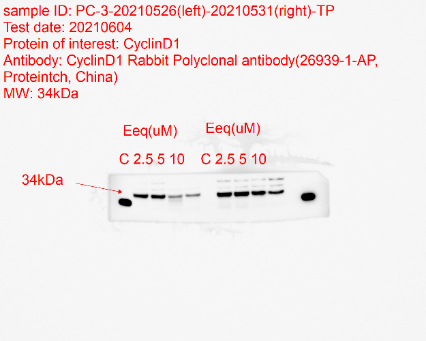


Sup. M.Fig.6 Original western blots images of CyclinD1.

## Fig.2G in our manuscript represents the analysis outcomes of the whole uncropped images in Sup.M.Fig.7.


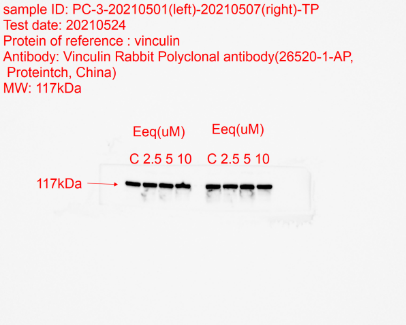

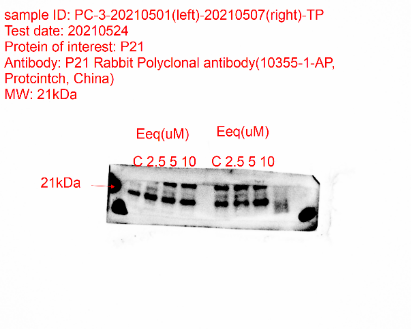


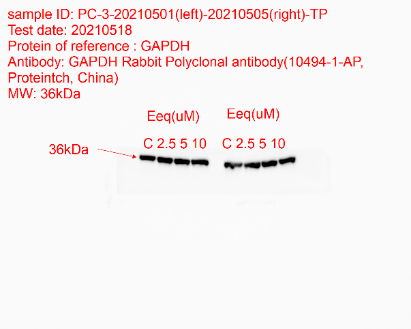

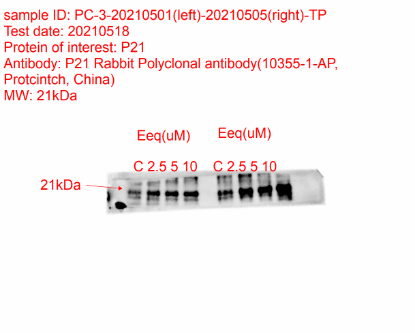


Sup. M.Fig.7 Original western blots images of p21.

## Fig.2H in our manuscript represents the analysis outcomes of the whole uncropped images in Sup.M.Fig.8.


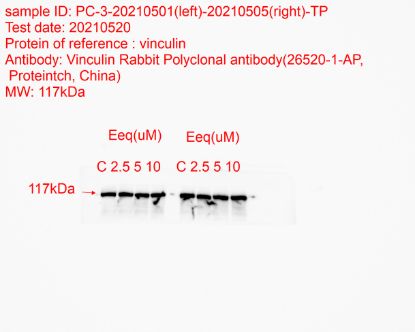

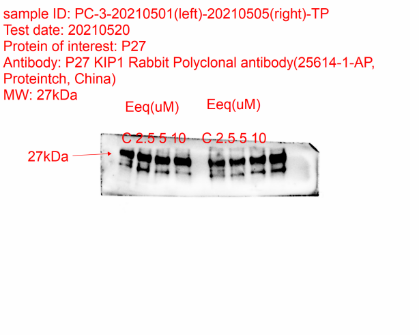


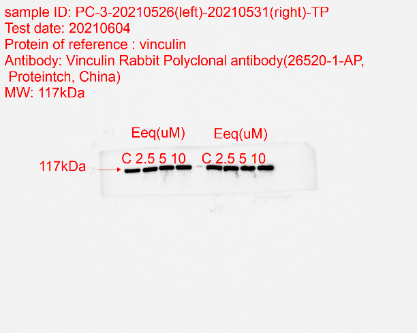

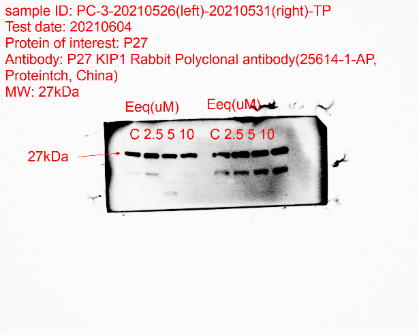


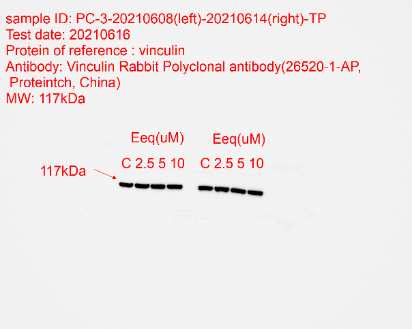

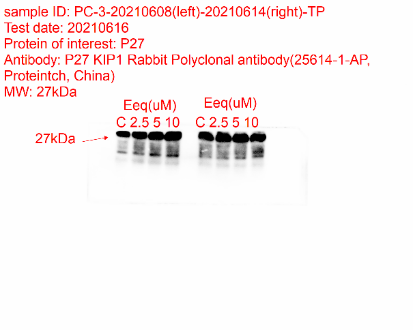


Sup. M.Fig.8 Original western blots images of p27.

## Fig.2I in our manuscript represents the analysis outcomes of the whole uncropped images in Sup.M.Fig.9.


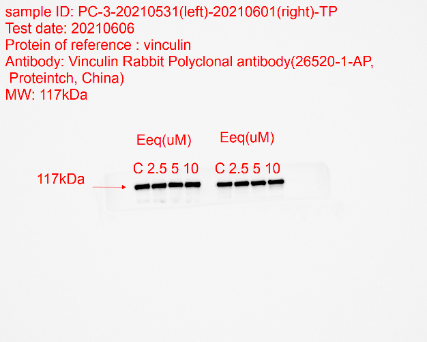

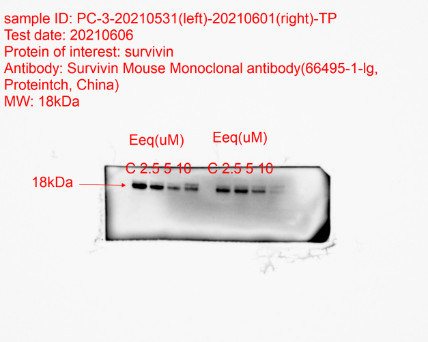


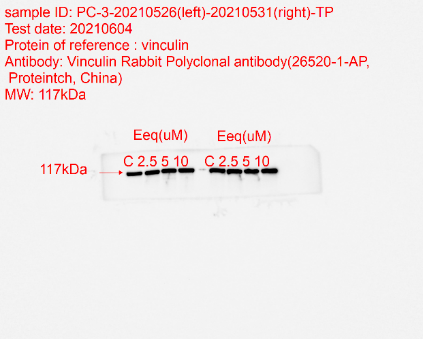

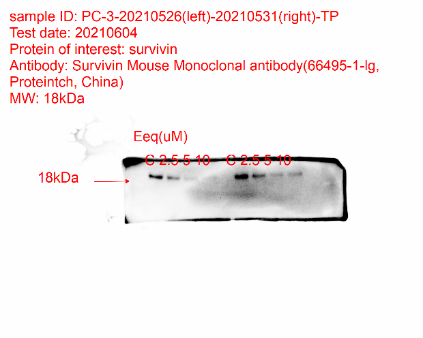


Sup. M.Fig.9 Original western blots images of Survivin.

## Fig.3B in our manuscript represents the analysis outcomes of the whole uncropped images in Sup.M.Fig.10.


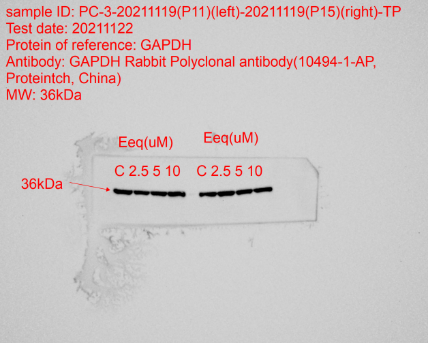

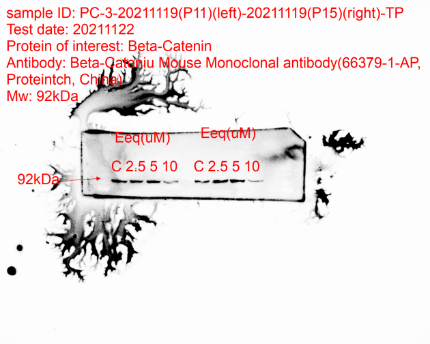


Sup. M.Fig.10 Original western blots images of β-catenin.

## Fig.3C in our manuscript represents the analysis outcomes of the whole uncropped images in Sup.M.Fig.11.


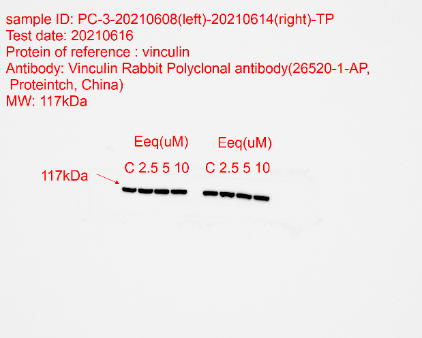

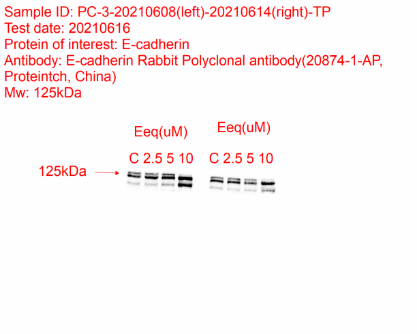


Sup. M.Fig.11 Original western blots images of E-cadherin.

## Fig.3D in our manuscript represents the analysis outcomes of the whole uncropped images in Sup.M.Fig.12.


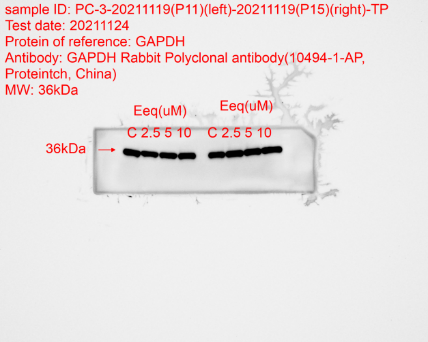

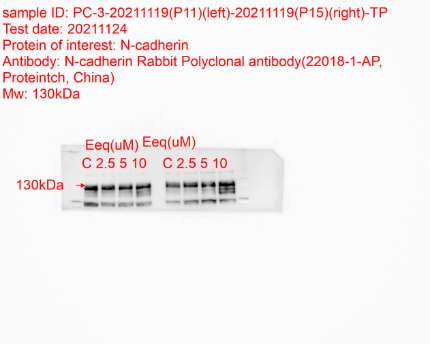


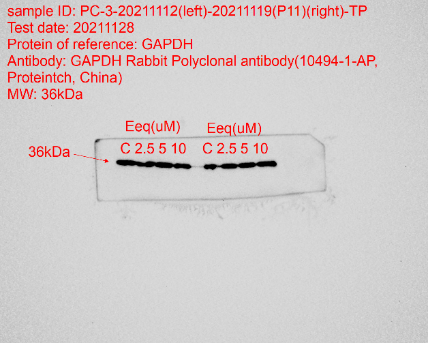

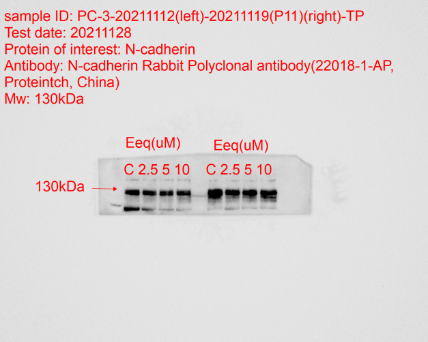


Sup. M.Fig.12 Original western blots images of N-cadherin.

## Fig.5A in our manuscript represents the analysis outcomes of the whole uncropped images in Sup.M.Fig.13.


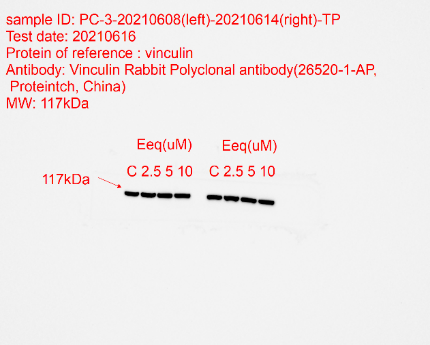

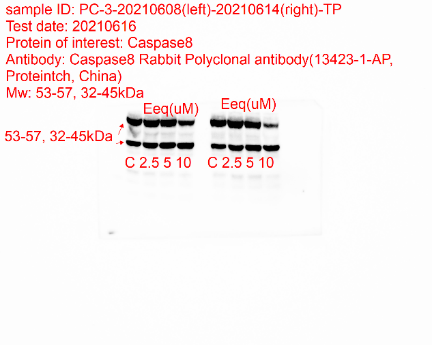


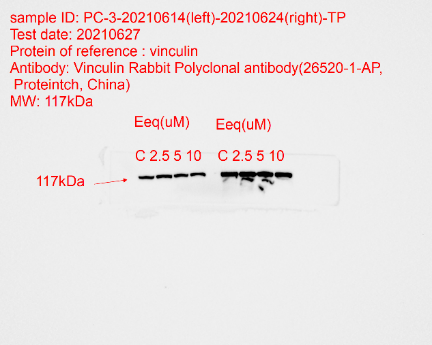

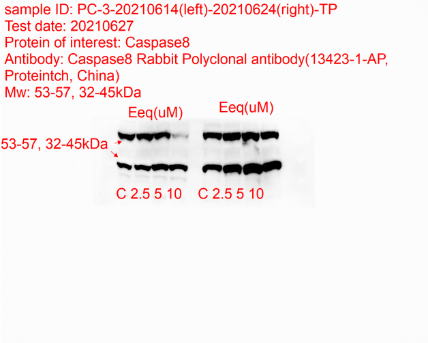


Sup. M.Fig.13 Original western blots images of Caspase 8.

## Fig.5B in our manuscript represents the analysis outcomes of the whole uncropped images in Sup.M.Fig.14.


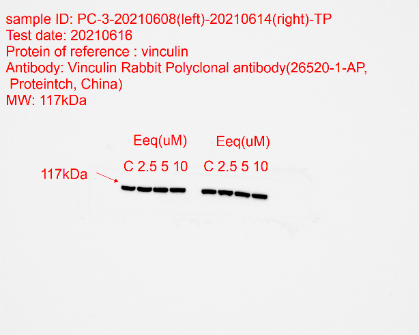

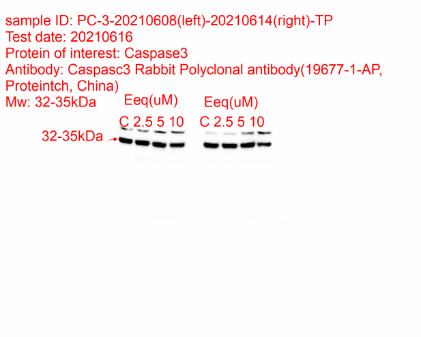


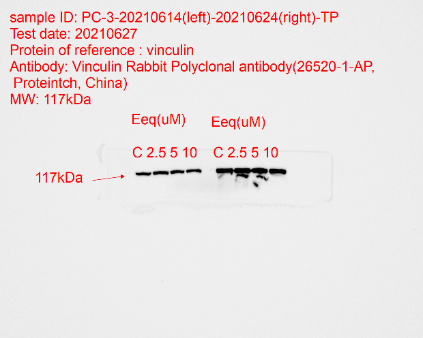

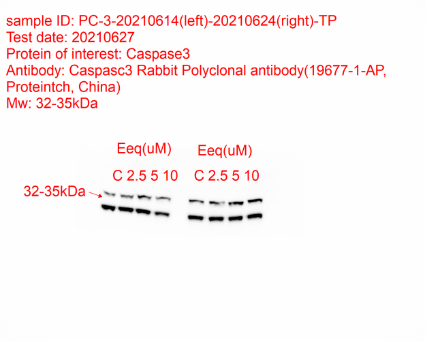


Sup. M.Fig.14 Original western blots images of Caspase 3.

## Fig.5D in our manuscript represents the analysis outcomes of the whole uncropped images in Sup.M.Fig.15.


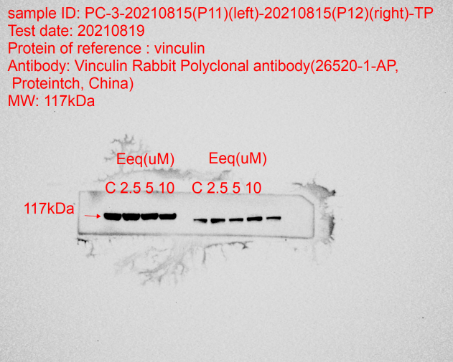

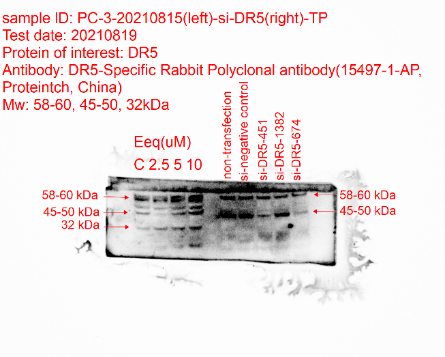


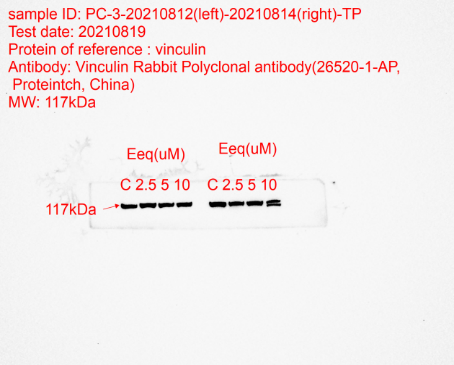

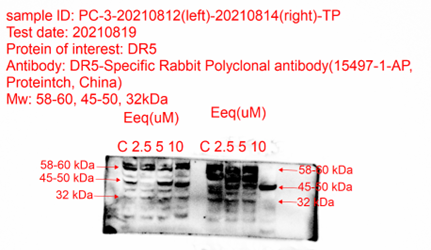


Sup. M.Fig.15 Original western blots images of DR5.

## Sup.fig.2F in our manuscript represents the analysis outcomes of the whole uncropped images in Sup.M.Fig.16.


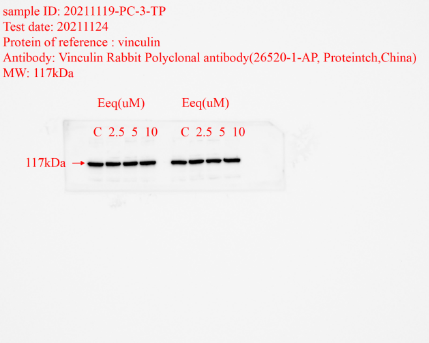

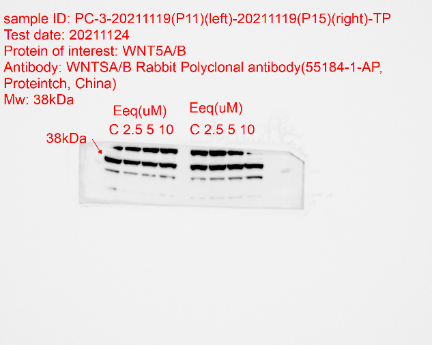


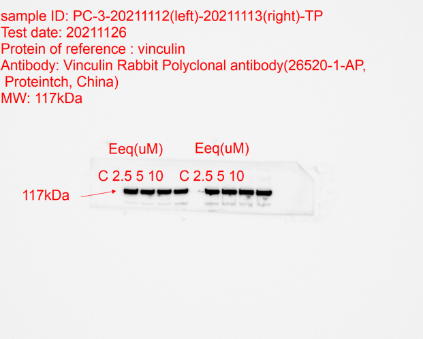

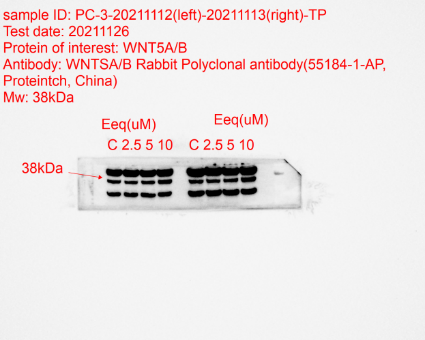


Sup. M.Fig.16 Original western blots images of WNT5A/B.

# Supplementary Figures


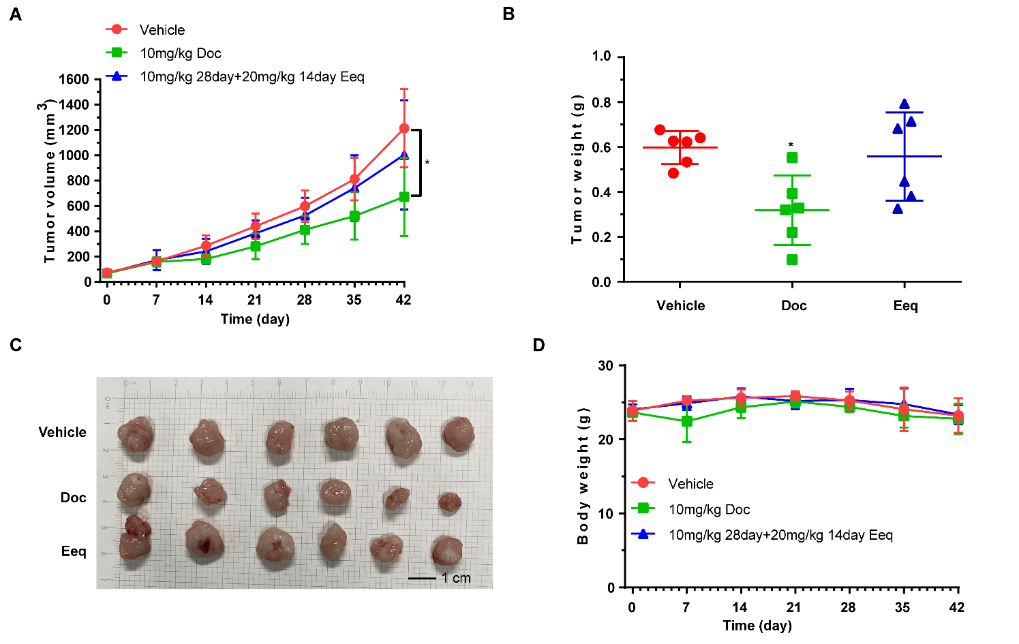


**Supplementary Figure 1.** 5'-epiequisetin (Eeq) showed weak tumor inhibition in mice at a dose of 10mg/kg and 20mg/kg. (A) Eeq reduced tumor volume in mice; (B) Tumor weight of mice; (C)Tumor size and appearance in mice; (D) Body weight of mice during administration. ^*^*P*<0.5, ^**^*P*<0.01 vs vehicle.


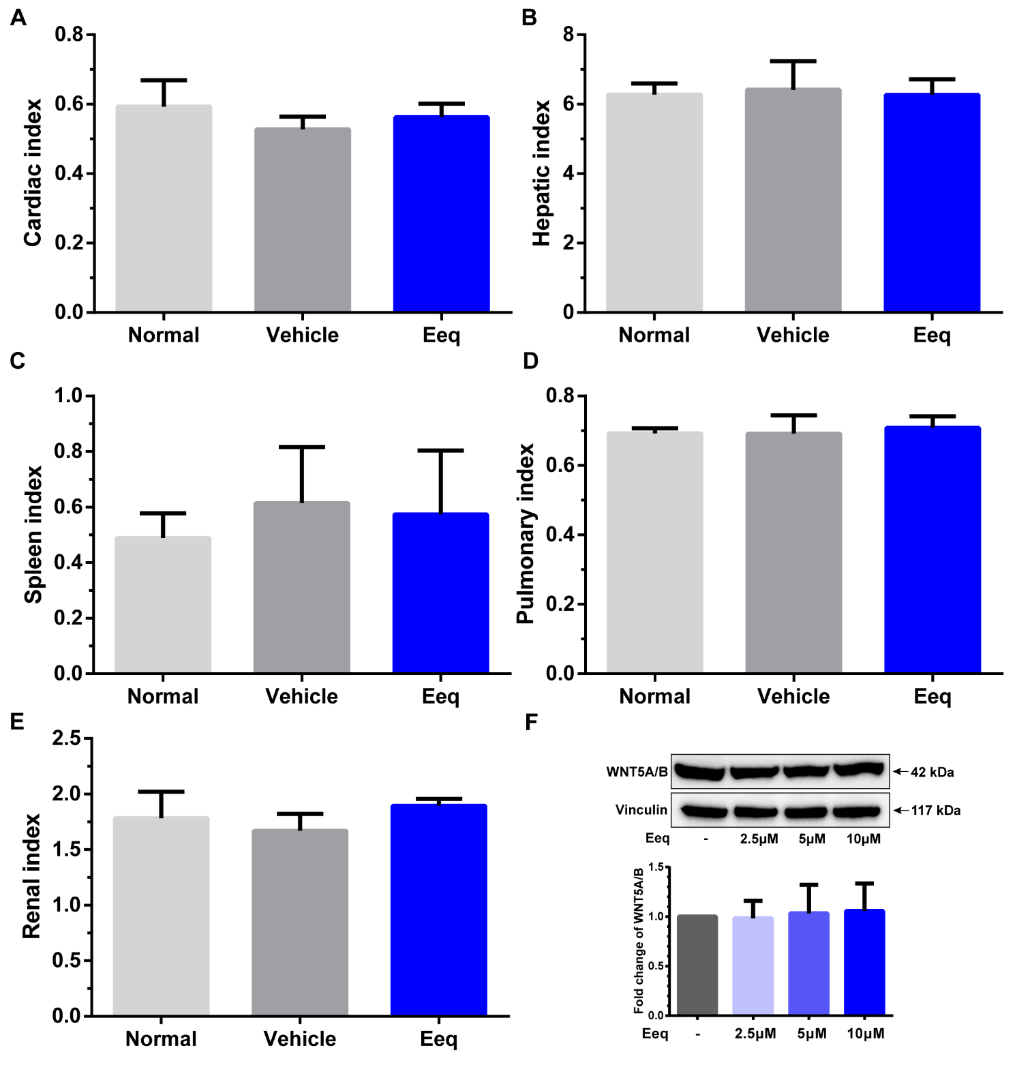


**Supplementary Figure 2.** 5'-epiequisetin (Eeq) showed no significant impact on organ index. (A) Cardiac index; (B) Hepatic index; (C) Spleen index; (D) Pulmonary index; (E) Renal index; (F) The protein level of WNT5A/B in PC-3 cells.
